# Improving Face Age Prediction by Using Multiple-Angle Photos

**DOI:** 10.34133/csbj.0019

**Published:** 2026-04-23

**Authors:** Botond Bárdos-Deák, Bence Király, Csaba Kerepesi

**Affiliations:** ^1^ Institute for Computer Science and Control (SZTAKI), Hungarian Research Network (HUN-REN), Budapest, Hungary.; ^2^ Institute of Mathematics, Budapest University of Technology and Economics, Budapest, Hungary.; ^3^ HUN-REN-SZTAKI-SU Rejuvenation Research Group, Office for Supported Research Groups (TKI), Hungarian Research Network (HUN-REN), Budapest, Hungary.

## Abstract

Face photo-based age provides a cost-effective and readily accessible tool for biological age studies. However, artificial intelligence-based face photo age models were usually trained on a single front-view photo per subject. Here, we hypothesized that face photo-based age prediction performance might be improved by using multiple photos of the same subject at the same time, captured from different angles. To test this hypothesis, we used an available dataset containing mugshots and developed age prediction models trained on (a) only front-view images, (b) only the side-view images, and (c) both front and side images. We found that accurate age prediction is possible using side photos despite the smaller facial area compared to front-facing photos [mean absolute error (MAE) = 3.1 years for the front-view and MAE = 3.7 years for the side-view images]. The age prediction performance further improved by using 2 images from one person at the same time, captured from 2 different angles, front and side (MAE = 2.88 years). We found that subjects who age faster based on front-view face photos generally age faster based on side-view face photos. We also found that side-view models handle the rotation of the face better compared to the front-view model. In summary, we showed that 2 photos of the face at different angles can slightly improve age prediction and may provide a more robust and better approximation of biological age compared to single photos, serving as a useful tool for personalized medicine, aging intervention, and rejuvenation studies. The models are available for academic research purposes at https://photoage.sztaki.hu/.

## Graphical Abstract

**Figure:**
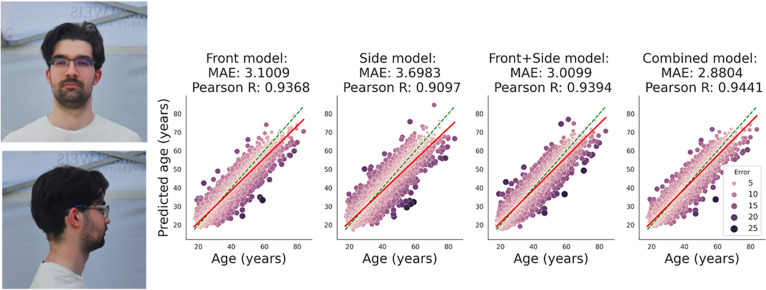

## Introduction

Aging is a major problem of the 21st century [1]. To find the solution is a big scientific challenge where bioinformatics and artificial intelligence (AI) can contribute by developing age prediction models (i.e., aging clocks) that provide accurate measurements of aging and biological age. In the last decade, different types of aging clock models were developed based on microscopic and macroscopic data for measuring the biological age of an individual [2–4]. Aging clocks not only predicted age accurately but also better predicted all-cause mortality than chronological age itself [5–10]. Hence, aging clocks suggest that some people age faster or slower than others, and accordingly, their biological age can be higher or lower than their chronological age. Age prediction models based on standard 2-dimensional (2D) face photos [11–13] and 3D face imaging [14–16] were developed too. In the current study, we focus on the simpler and standard 2D face photo-based aging clock models that may provide a low-cost, readily accessible biological age approach. Recently, we showed that face photo-based age acceleration calculated by AI is predictive of all-cause mortality [17]. Others showed that face photo-based age predicts mortality of cancer patients better than chronological age [18]. These results suggest that face photo-based age approaches biological age better than chronological age and may provide a cost-effective and readily accessible tool for personalized medicine and rejuvenation studies. However, face photo-based aging clocks typically were trained on a single photo per person, usually captured from the front view. As using multiple images instead of one showed benefits in face recognition [19,20], here we investigate whether face photo-based age prediction performance can be improved by using 2 photos of the same person at the same time, captured from different angles, one from front-view and one from side-view. We hypothesize that 2 photos at different angles can improve age prediction and may provide a better tool for approaching biological age.

## Results

### Improved AI-based face age prediction using both front-view and side-view facial photos

**Fig. 1.**
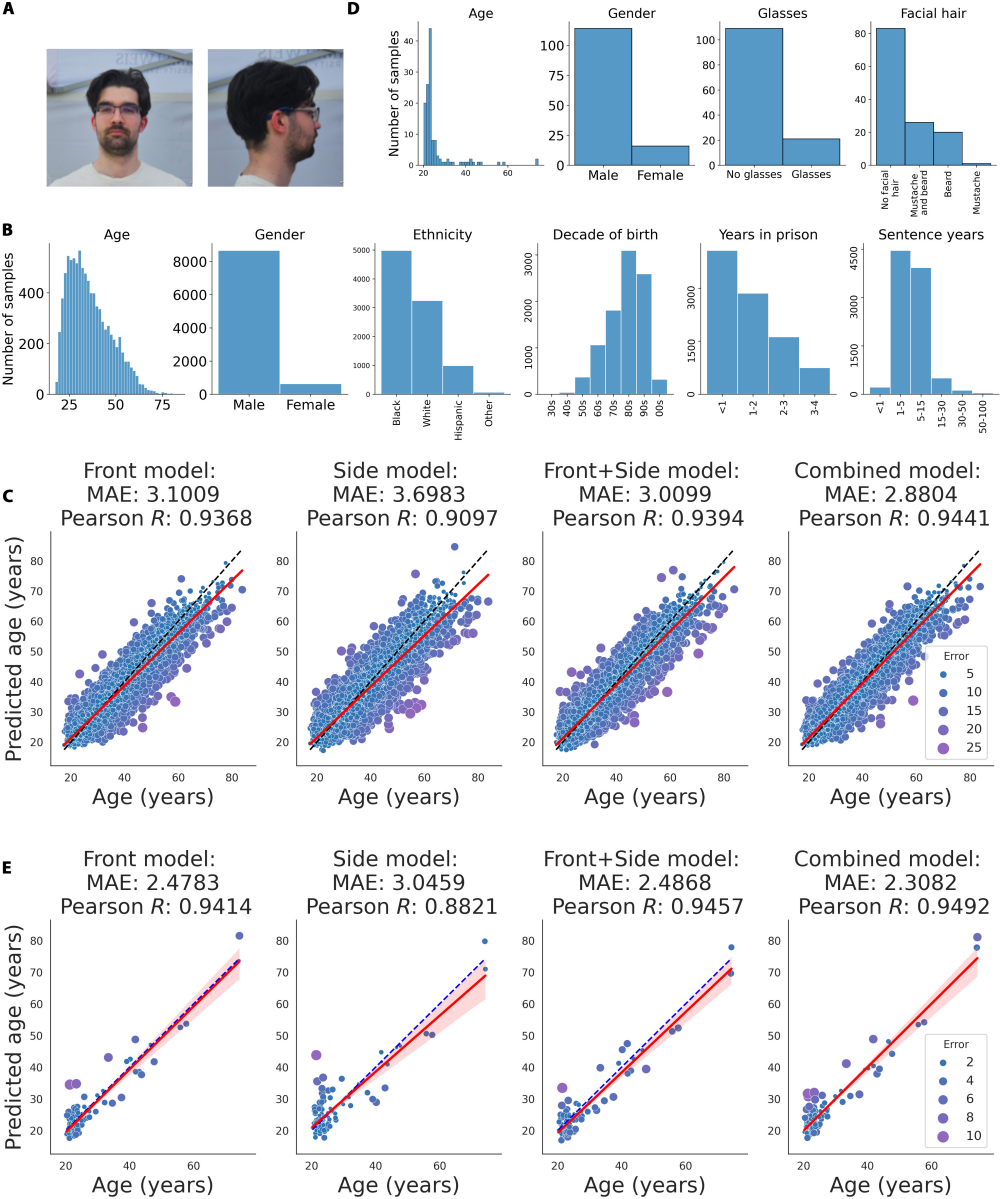
Improved face age prediction using both the front-view and side-view face photos. (A) Illustrative mugshot of the first author of this study (the training and testing sets did not include these 2 pictures). (B) Distribution of the attributes of the testing set of the Prisoner dataset. (C) Age prediction performances of the face photo age models using the testing set of the Prisoner dataset. (D) Distribution of the SCface dataset attributes. (E) Prediction performances of the face photo age models on the external dataset, SCface.

We tested whether face photo-based age prediction performance can be improved by using 2 photos of the same person at the same time captured from different angles (Fig. 1A). For this purpose, we collected available data (i.e., paired front-view and side-view face photos) of 54,295 Illinois state prisoners (the “Prisoner dataset” hereafter) and split the high-quality mugshots of the Prisoner dataset into training, validation, and testing sets by the subjects. Then, we developed 3 separate models trained on (a) front-view (Front model), (b) side-view (Side model*)*, and (c) concatenated front-view and side-view mugshots (Front + Side model). We developed another model by combining the predictions of the Front model and Side models (Combined model). We trained the models on the training set, optimized using the validation set, and tested the final models on the testing set, predicting the age of the subjects. The Front model was more accurate than the Side model [mean absolute error (MAE) = 3.1 versus 3.7 years]. However, using both images of the face, the age prediction performance further improved (MAE = 3.01 years by the Front + Side model and MAE = 2.88 years by the Combined model; Fig. 1B and C and Table S1).

We also tested the age prediction models on an external dataset, SCface [21], containing high-quality photos of 130 subjects taken from different angles (Fig. 1D). We applied the Front model on the front-view photos, the Side model on the side photos, as well as the Front + Side and Combined models on both photos (Fig. 1E and Table S1). Again, the Front model was more accurate than the Side model (MAE = 2.48 versus 3.05 years), and the Combined model performed the best (MAE = 2.31 years). In summary, these results show that the models generalize very well in a different population.

**Fig. 2.**
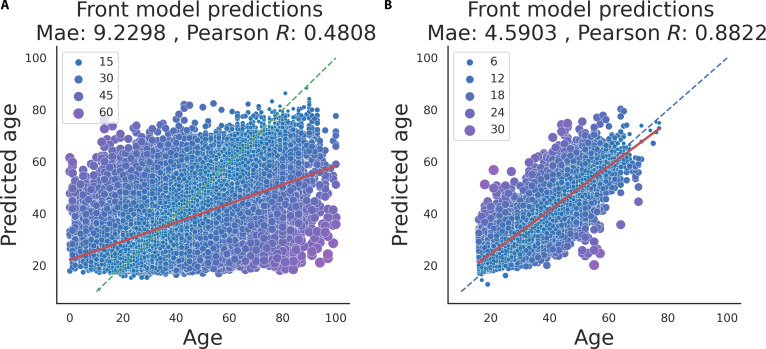
Testing the Front model on further independent external datasets. (A) Age prediction of the Front model on the IMDB-Clean dataset, containing in-the-wild photos (typically non-mugshot) of famous people. (B) Age prediction of the Front model on the MORPH-2 mugshot dataset.

Furthermore, we tested the Front model on 2 other independent external datasets, the IMDB-Clean and MORPH-2 (Fig. 2A and B). The model generalized well in the MORPH-2, containing frontal-view mugshots (MAE = 4.5903 years, and *r* = 0.8822); however, it did not perform well in the “in-the-wild” (i.e., not ID-card style studio photos) dataset of famous people of the IMDB-Clean database (MAE = 9.23, and *r* = 0.48).

Overall, we conclude that accurate age prediction is possible using side photos despite the smaller facial area compared to front-facing photos. The age prediction performance further improved by using 2 images from one person at the same time, captured from different angles, front and side.

### The age acceleration based on front-view face photos correlated with the age acceleration based on side-view face photos

**Fig. 3.**
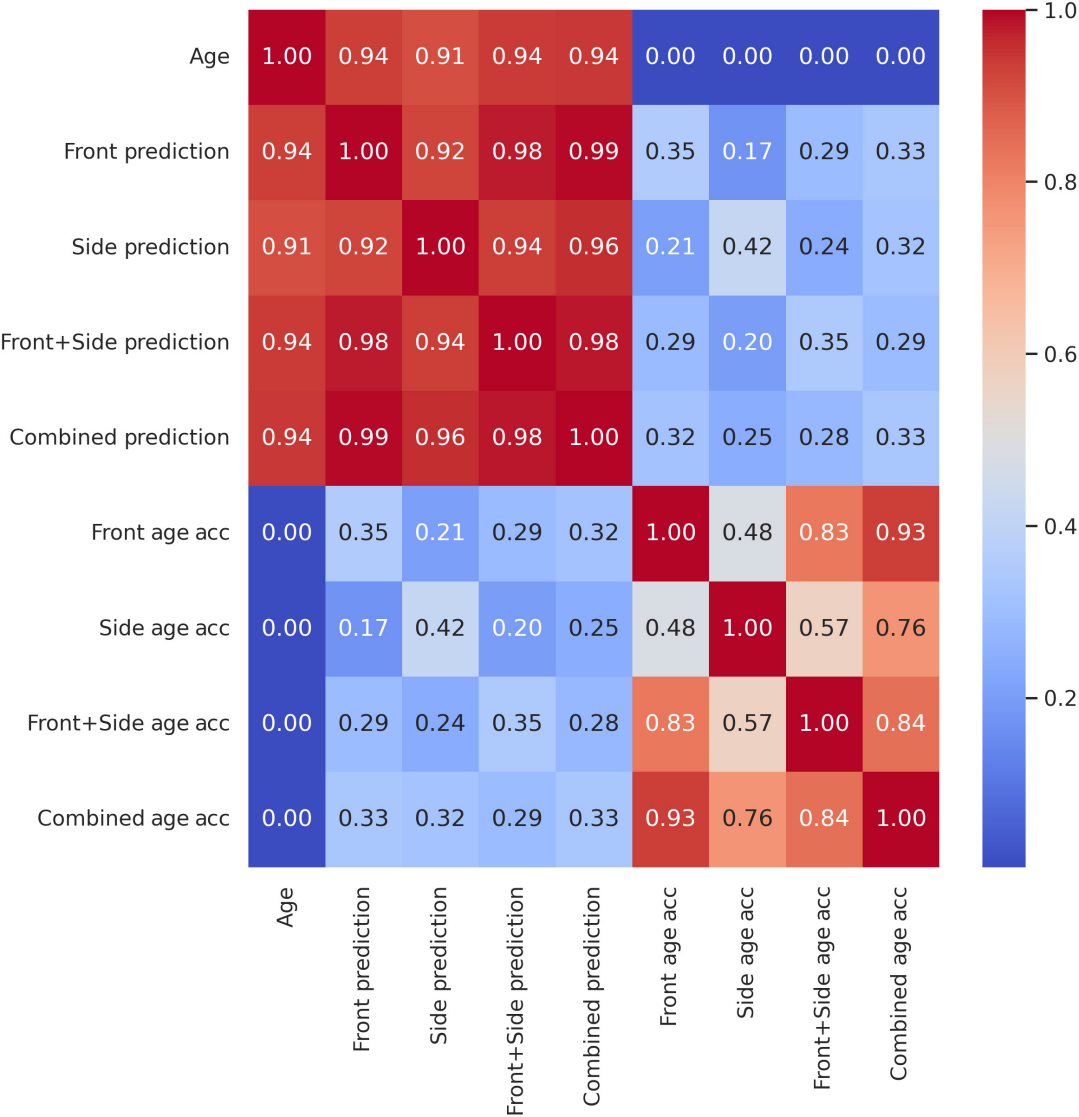
All versus all correlation analysis of age, predicted age, and age acceleration of the 4 face photo-based aging clock models on the testing set of the Prisoner dataset.

**Fig. 4.**
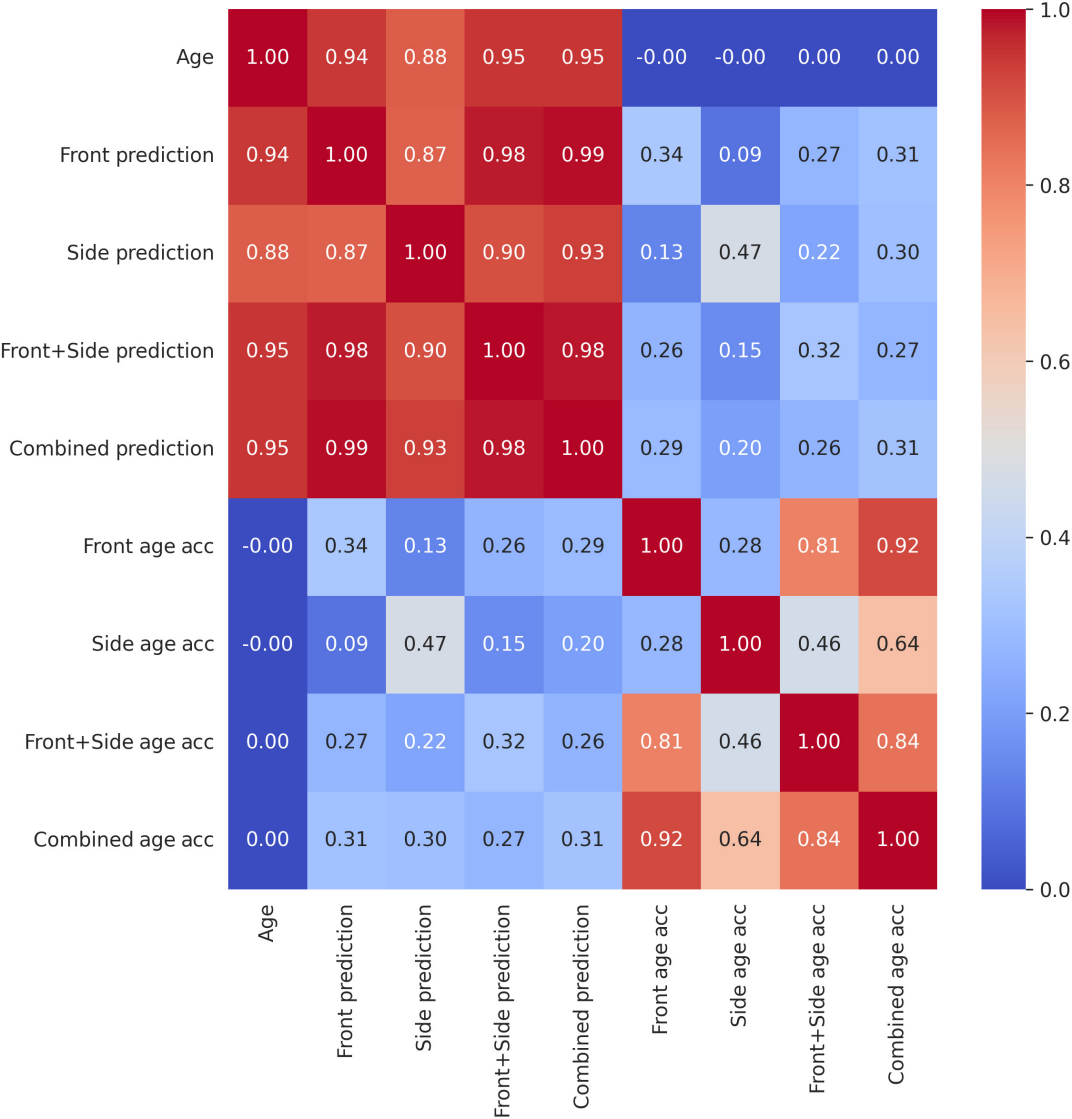
All versus all correlation analysis of age, predicted age, and age acceleration of the 4 face photo-based aging clock models on the SCface dataset.

We examined the correlations between the different models applied on the test set of the Prisoner dataset (Fig. 3). The age prediction of the models correlated very well with each other (*r* > 0.91). Age acceleration, which is the deviation of the predicted age from the trend, did not correlate with age, allowing the comparability of different age groups in age acceleration analyses. However, the age acceleration of the Front model significantly correlated with the age acceleration of the Side model (*r* = 0.48), meaning that subjects that age faster based on their frontal face photo generally age faster based on their side-view photo too (Fig. 3). The age acceleration of the Front model significantly correlated with the age acceleration of the Side model (*r* = 0.28) in the external test set (the SCFace dataset) too (Fig. 4).

We also observed that the correlation of age accelerations between the Front and Front + Side model was higher than the correlation of age accelerations between the Side and Front + Side model. The same was true for the Combined model. We concluded that while age prediction using side-view face photos showed comparable accuracy with front-view face photos, the frontal-view photo became more important for the 2-image models.

### The Side model handles the rotation of the face better compared to the Front model

**Fig. 5.**
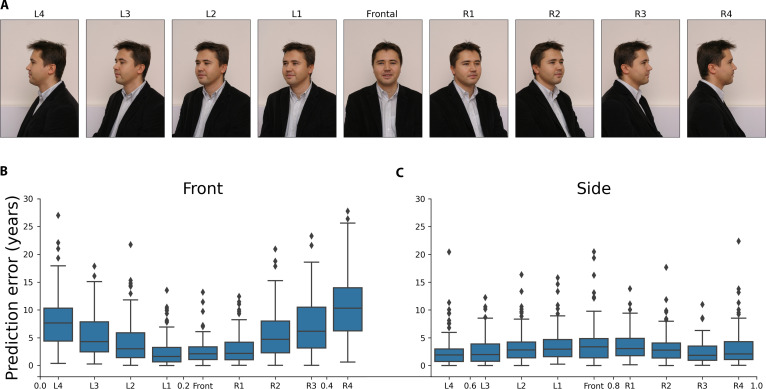
(A) Example images from the SCface dataset (adapted from the website of the SCface database; https://www.scface.org/) [21]. Image is not covered by the CC BY 4.0 license of this article. (B) Absolute error of the Front model applied on different face angles on the SCface dataset. (C) Absolute error of the Side model applied on different face angles on the SCface dataset.

The SCface dataset contains 9 pictures from different angles of every individual from left to right side in equal steps by 22.5°. The different angles are labeled as “frontal”, “L1” to “L4”, and “R1” to “R4”, as the angle increases from the frontal picture (Fig. 5A). When we tested the Front model on the SCface dataset in all available angles, we observed that the age prediction became less accurate as the angle increased starting from the front-facing image (Fig. 5B and C and Table S2). Interestingly, the Side model handled the rotation of the face better, providing more consistent results (the MAE was between 2.47 and 10.48 for the Front model and between 2.35 and 3.87 for the Side model). Furthermore, rotating the face provided more accurate age prediction at almost every angle, except for the front-facing and slightly rotated (i.e., R1 and L1) images. As the angle decreased, the MAE of the Side model increased only to a small extent, much less compared to the Front model.

Then, we tested the Side model exclusively on the front images of the Prisoner, IMDB Clean, and MORPH-2 datasets (Table S3) and observed slightly worse results compared to the Front model. In contrast, when we tested the Front model exclusively on side-view photos, the Front model performed much worse than the Side model (Table S4). In summary, the Side model performed much better on the frontal-view face photos than the Front model on the side-view face photos. An explanation of this observation can be that the Side model may focus on features that are completely covered in all-angle photos, while the Front model, similarly to our previous FamousAge model [17], may focus on the nose–mouth areas that are not completely covered in the side photos.

### Gender and ethnicity bias analysis

As the Prisoner dataset had a huge gender bias (93.1 % males and 16.9% females), we conducted a gender bias analysis in the different testing datasets (Table S5). While the prediction accuracy was worse for females compared to males in the testing set of the Prisoner and SCFace datasets, it remained remarkably accurate (MAE = 3.62 and 3.21 years, respectively, for the combined model). Interestingly, the models showed very small gender differences in MAE for the MORPH-2 and IMDB-Clean datasets. We also calculated the MAE of genders within the different ethnic groups for the testing set of the Prisoner dataset (Table S6). The group “other” (i.e., other than white, black, and Hispanic) females and white males showed the lowest MAE (2.26 and 2.75 years, respectively), while black females and “other” females showed the highest MAE (4.54 and 4.27 years).

We also performed an ethnicity bias analysis, as follows. We selected 4,615 frontal-view photos from each of the 3 largest ethnic groups: black, Hispanic, and white. Then, we trained models for each of the 3 specific ethnicity labels, and a fourth for the merged dataset containing 3 × 4,615 images. In all cases, the model trained on the combined dataset was more accurate than the ethnic-specific models tested on their respective ethnic groups and other ethnic groups. While the larger sample size of the combined dataset can affect the results, we conclude that a combined model works well on all major ethnicities of the dataset (Table S7).

### The mouth, nose, and eye are the most important areas in the face photo-based age models

**Fig. 6.**
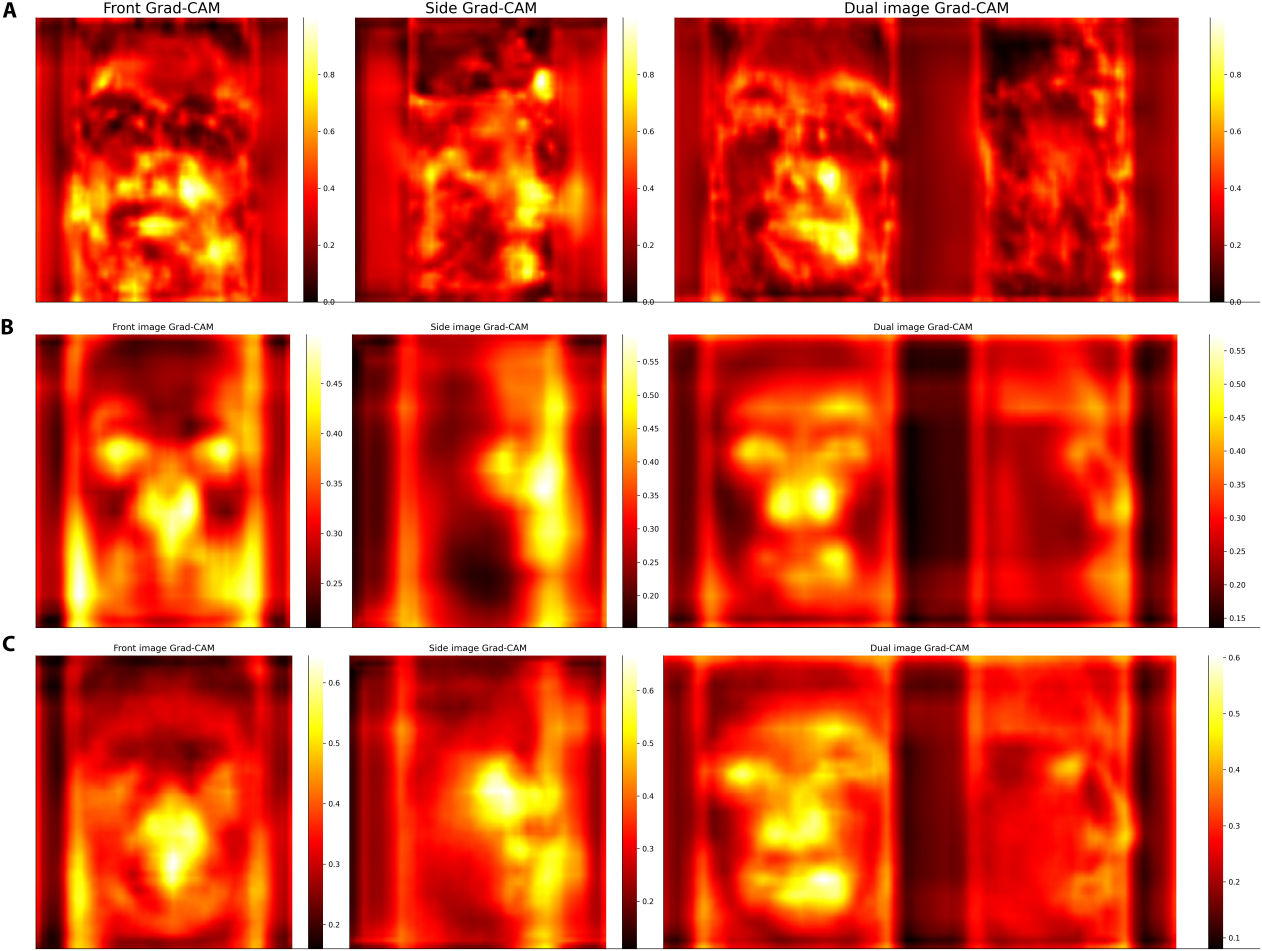
The mouth, nose, and eye are the most important areas for the face photo-based age models. (A) Grad-CAM values calculated for the cropped face photos of Fig. 1A. The chronological age was 23.7 years. The predictions by the Front, Side, and Front + Side models were 25.28, 26.55, and 25.42 years, respectively. (B) Average Grad-CAM values of the testing dataset of the Prisoners dataset. (C) Average Grad-CAM value on the SCface dataset.

For model explanation, we used the Grad-CAM method [22]. We calculated the average Grad-CAM values aggregated for all layers and all pictures for an example mugshot (Fig. 6A), the testing set of Prisoner dataset (Fig. 6B), and for the SCface dataset (Fig. 6C). The heatmaps visualize the importance of the pixels in the model predictions. The nose, mouth, and eyes were among the most important areas for all models. These results are consistent with previous frontal image-based age prediction models [17,23]. The Grad-CAM analysis also showed that the frontal face image is much more important than the side image for the Front + Side model (Fig. 6A to C). This observation is consistent with our finding based on age accelerations (see above).

## Discussion

Here, we developed deep learning age prediction models using mugshots (i.e., frontal and side-view face photos). We conclude that it is possible to predict age from side-view face photos with almost the same accuracy as using front-view face photos. We also showed that 2 photos at different angles (front and side) improved age prediction and may provide a better tool for measuring biological age. Recently, it was shown that age acceleration based on single photos of the face predicts all-cause mortality [17] and mortality of cancer patients [18]. In the future, it would be useful to test if AI-based face photo age correlates with other biological aging markers.

So far, only a few studies have reported age predictions based on multimodal data (i.e., multiple types of measurements of the same individual at the same time). Wang et al. [16] trained age prediction models using 3D facial, tongue, and retinal images from healthy subjects and demonstrated that using a fusion of the 3 image modalities achieved the most accurate predictions. Recently, we developed aging clocks using the methylation and gene expression features at the same time, and found that the most accurate models tended to use methylation features exclusively [24]. Future studies need to explore the benefits of the different data modalities in measuring biological age.

Multi-view learning (i.e., learning on data from different modalities, sources, spaces, and other forms, but with similar high-level semantics) has been receiving increasing attention [25]. In computer vision, it was shown that a multi-view 3D face reconstruction algorithm using multiple images of the same subject can improve the face identification accuracy compared to single-image approaches [26]. An early study explored the multi-view facial expression recognition using different angles [27]. A multi-view face detection method that did not require pose/landmark annotation was able to detect faces in a wide range of orientations using a single model based on deep convolutional neural networks [28]. Using multiple images of the same individual instead of one improved human face matching accuracy in some experiments [19,20]; however, other experiments showed no benefits reported from exposure to arrays comprising a frontal and a profile view image [29] or when matching a live person to a 4-image array compared to a single image [30].

The Prisoner models were trained on mugshots of a very special population (mostly male prisoners with a diverse ethnicity), yet it generalized very well on the mugshots of the SCface database with mostly male Caucasian students, professors, and employees with a slightly better performance than in the original testing set of the Prisoner database (MAE was between 2.3082 and 3.0459 years, and *r* was between 0.8821 and 0.9492). Additionally, we tested the frontal model in the MORPH-2 database containing front-view mugshots collected from the general (non-prisoner) population, and it reached a high accuracy with 4.59 MAE and *r* = 0.88. However, the frontal Prisoner model did not perform well on the IMDB-Clean dataset, which contains in-the-wild (typically not mugshot photos) of famous people. While the mugshot datasets had images looking in the same direction and in very similar light conditions, the IMDB-Clean is a dataset containing images with various light and pose settings captured in various situations. As we saw in Fig. 5, the accuracy of the Front model decreased as the head was rotated. It can explain the pure accuracy of our Front model in the IMDB-Clean dataset. Additionally, as the IMDB-Clean was automatically collected and annotated, it may have some annotation errors (i.e., wrong ages). In summary, the Prisoner model generalizes well in mugshot datasets from various population; however, it may not be optimal for in-the-wild datasets.

Face photo-based age can be the cheapest and fastest approximation of biological age. In the near future, even more developed versions can emerge, and it can be a useful tool in personalized medicine when doctors can consider biological age, among other factors, in treatments. It will be beneficial to integrate face photo-based biological age approximations into the healthcare system and treat patients according to their biological age instead of their chronological age. It was shown that face photo-based age acceleration determined by AI models is a risk factor of all-cause mortality [17] and cancer [18] and can be useful for disease prognostication. Here, we propose using 2 photos in different angles instead of one frontal photo to provide a more accurate and robust measurement of face photo age in personalized medicine. The gold-standard measurements of biological age nowadays are DNA methylation aging clocks; however, they are still not perfect. Epigenetic studies and other expensive measurements of biological age, such as transcriptomics, proteomics, and magnetic resonance imaging scans, can be easily combined with face photo age measurements to reach a more robust and accurate approximation of biological age. For example, our face photo-based age methods can be applied as a complementary measurement of rejuvenation experiments, as an ideal aging intervention should be reflected in the appearance of the subject too. Face photo-based age is also suitable to explore longitudinal aging dynamics, as it is easy to measure in different time points of the same subject.

In summary, we showed that 2 photos of the face at different angles can slightly improve age prediction and may provide a more robust and better approximation of biological age compared to single photos, serving as a useful tool for personalized medicine, aging intervention, and rejuvenation studies.

## Methods

### The Prisoner dataset

We used the publicly available (license CC0: Public Domain) Kaggle IDOC (Illinois Department of Corrections) mugshot dataset (https://www.kaggle.com/datasets/elliotp/idoc-mugshots). The dataset contained mugshots (i.e., photographic portraits of each subject from the shoulders up with a plain background) with a front-view and a side-view photo of each individual who was in prison at the end of 2018. The mugshots are taken at the date of admission to the prison, which can be earlier than 2018, but the photos are updated periodically. To decrease the chance that the mugshots were updated since the date of admission, we used only the mugshots of individuals with an admission date between 2015 and 2018. We used additional mugshots directly downloaded from the publicly available Illinois Prison Population Data Sets (https://idoc.illinois.gov/reportsandstatistics/populationdatasets.html) of individual who were in prison in July of 2024, and their admission dates were between 2021 and 2024. According to the IDOC website, the dataset is provided for the general public to facilitate research (https://idoc.illinois.gov/reportsandstatistics.html). Using the available annotations, we assigned ages to the photos by the formula Age = Admission date − Birth date. In summary, we collected high-quality mugshots of 51,149 individuals, 47,642 of males, and 3,507 of females. The distribution of ethnicity was 63 of American Indian, 156 of Asian, 116 of biracial, 27,617 of black, 5,638 of Hispanic, 17,500 of white, and 59 of unknown ethnicity. These ethnicity labels were provided by the dataset; we used them without any change. The youngest age was 17 years, and the oldest age was 83 years.

### Training and testing of the age prediction models

We split the data into training, validation, and testing sets by using 0.6, 0.2, and 0.2 ratios, respectively, and removed those pictures when multiple pictures were taken of one person. After this, the training set contained 32,577 images, the validation set contained 9,281 images, and the test set contained 9,291 images. All of the images were cropped using the Retinaface face detector [31] followed by padding to a square and resized to 224 × 244 pixels. We finetuned a RES-NET 50 model that was pretrained using IMAGENET1K_V2 and modified its final layer to one neuron. We used the ADAM optimizer and the mean squared error loss function with reduce on plateau learning rate scheduler that decreases the learning rate if the performance does not improve over a given number of epochs, as well as early stopping. For data augmentation, we used normalization, random affine transformations, grid distortion, random brightness, and contrast change. We trained separate models using (a) only front face photos (Front model), (b) only side-view face photos (Side model), and (c) both front- and side-view face photos (Front + Side model). We constructed another model by combining the predictions of the Front and the Side models by using an ordinary least squares method on the validation set. Finally, we got the following formula: Combined model predicted age = −0.7285 + 0.7467 ∗ (Front model predicted age) + 0.2951 ∗ (Side model predicted age), and after this, we tested the Combined model on the testing set of the Prisoner dataset and the SCface datasets without any changes in the formula.

### External testing datasets

We used the SCface dataset (https://www.scface.org/) for external testing, containing high-quality mugshots of 130 people (116 males and 14 females) captured from 9 different angles. The database contained additional annotations about gender, beard, moustache, and glasses.

We also used another independent mugshot dataset (containing only frontal-view mugshots), the MORPH-2 (https://www.kaggle.com/datasets/chiragsaipanuganti/morph) [32], for an additional external testing of our Front model. The MORPH-2 dataset contained 55,134 mugshots of ~13,000 subjects; 14.14% of the images were from females. The subject age was between 16 and 77 years. The dataset had an unbalanced gender distribution with 36,832 of males and 5,757 of females and ethnicity distribution with African: 42,589, European: 10,559, Hispanic: 1,769, Asian: 154, and “Other”: 63.

We tested the Front model on the IMDB-Clean dataset as well (https://github.com/yiminglin-ai/imdb-clean?tab=readme-ov-file), which was an “in-the-wild” dataset, meaning that the photos were taken in real-life scenarios [33,34]. This dataset consisted of 221,055 images; 42.85% of the individuals were females.

### Model explanation

We used the Grad-CAM Python package [35] to visualize the important regions of the models. We used all layers of a particular model and calculated the Grad-CAM value of each pixel of each image. Then, we calculated the mean of the pixels and visualized it as a heatmap. All parameters and settings were the same as in the model testing.

### Statistics

To measure the performance of our models, we used *R*^2^ score, Pearson R, MAE, and cumulative score 3 CS_3_(%) (i.e., the percentage of the predictions had less than 3 years of absolute error). We used *P* values to determine whether the correlation is statistically significant. Significance levels are marked using asterisks, ns: *P* > 0.05, *: 0.01 < *P* ≤ 0.05, **: 0.001 < *P* ≤ 0.01, ***: 0.0001 < *P*≤ 0.001, ****: *P* ≤ 0.0001.

## Data Availability

The Python code for testing the models is available on GitHub: https://github.com/bdbotond/dual_face_age_pred. Furthermore, we have a web application at https://photoage.sztaki.hu where the models are available for academic research purposes. As the models are among the first attempts using face photos for biological age assessment, it is not recommended to use them in clinical practice yet. However, we believe that it can be useful for scientists to accelerate this direction of research. We do not store any images to ensure privacy.
