# Supplementary material for: Improving Face Age Prediction by Using Multiple-Angle Photos
Source: Comput Struct Biotechnol J. 2026 Apr 23;35(1):0019. doi: 10.34133/csbj.0019 (PMC13104633; doi:10.34133/csbj.0019)
Supplement: Supplementary 1 — Fig. S1 Tables S1 to S7 [file csbj.0019.f1.docx]

**SUPPLEMENTARY FIGURES AND TABLES**

**
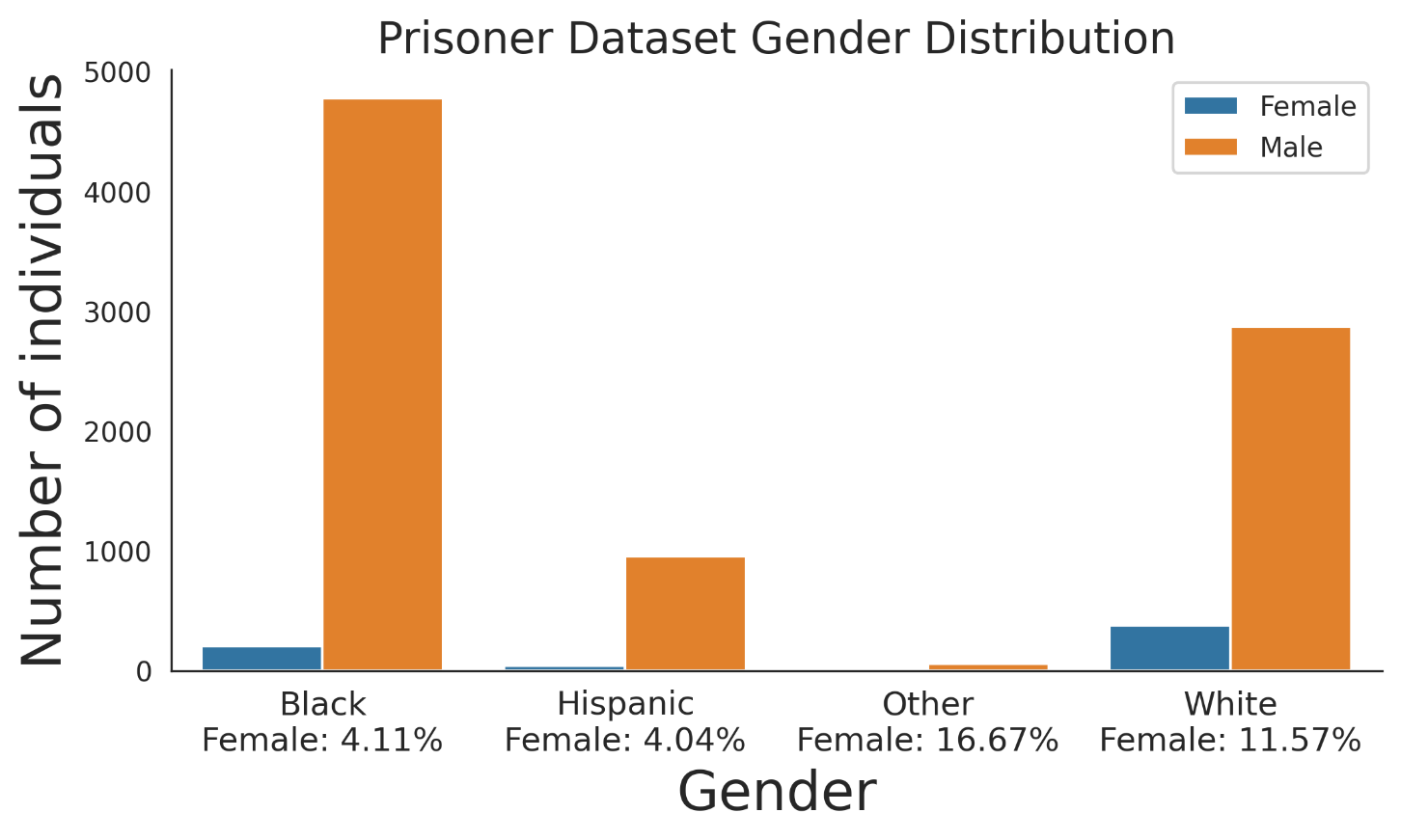
**

**Supplementary Figure S1. Gender distribution of the different ethnic groups in the Prisoner dataset.**

**Supplementary Table S1. Prediction performances of the models in the testing datasets**

| Dataset | *R*^2^ Score | Pearson R | Pearson R CL 95 | Pearson R CU95 | MAE | *CS*_3_(%) |
| --- | --- | --- | --- | --- | --- | --- |
|  | | | | | | |
| Prisoner front images | 0.87 | 0.94 | 0.9343 | 0.9392 | 3.11 | 0.8 |
| SCface front images | 0.87 | 0.94 | 0.9181 | 0.9583 | 2.48 | 0.93 |
| MORPH-2 | 0.72 | 0.88 | 0.8802 | 0.8841 | 4.59 | 0.62 |
| IMDB-Clean | 0.14 | 0.48 | 0.4776 | 0.484 | 9.23 | 0.37 |
|  | | | | | | |
| Prisoner side images | 0.82 | 0.91 | 0.9061 | 0.9131 | 3.69 | 0.73 |
| SCface side images | 0.76 | 0.88 | 0.8370 | 0.9152 | 3.05 | 0.82 |
|  | | | | | | |
| Prisoner two images | 0.88 | 0.93 | 0.9369 | 0.9417 | 3.01 | 0.82 |
| SCface two images | 0.89 | 0.95 | 0.9240 | 0.9614 | 2.49 | 0.89 |
|  | | | | | | |
| Prisoner two images | 0.89 | 0.94 | 0.9418 | 0.9462 | 2.88 | 0.83 |
| SCface two images | 0.89 | 0.94 | 0.9288 | 0.9638 | 2.31 | 0.91 |

**Supplementary Table S2. MAE of the Front model and the Side model from different angles in the SCFace dataset**

| Angle | MAE (Front model) | MAE (Side model) |
| --- | --- | --- |
| L1 | 2.47 | 3.56 |
| L2 | 4.18 | 3.3 |
| L3 | 5.27 | 2.62 |
| L4 | 8.18 | 2.47 |
| R1 | 2.98 | 3.5 |
| R2 | 5.74 | 3.14 |
| R3 | 7.02 | 2.35 |
| R4 | 10.48 | 3.05 |
| frontal | 2.48 | 3.87 |

**Supplementary Table S3. Side model results compared to the Front model results on frontal images**

|  | Side model | Front model |
| --- | --- | --- |
| Dataset | MAE | MAE |
| Prisoner front images | 6 | 3.14 |
| SCface front images | 3.87 | 2.48 |
| MORPH-2 | 7.21 | 4.59 |
| IMDB-Clean | 10.17 | 9.23 |

**Supplementary Table S4. Front model results compared to the Side model results on side images**

|  | Front model results on the side images | Side model results on the Side images |
| --- | --- | --- |
| Dataset | MAE | MAE |
| Prisoner front images | 8.93 | 3.72 |
| SCface front images | 10.48 | 3.05 |

**Supplementary Table S5. Gender and ethnicity bias analysis**

| Prisoner dataset | | | | |
| --- | --- | --- | --- | --- |
| Gender | Front model | Side model | Front + Side model | Combined model |
| Female | 4.06 | 4.59 | 3.77 | 3.62 |
| Male | 3.07 | 3.67 | 2.99 | 2.86 |
| SCface dataset | | | | |
| Gender | Front model | Side model | Front+Side model | Combined model |
| Female | 3.24 | 5.06 | 3.33 | 3.21 |
| Male | 2.37 | 2.76 | 2.37 | 2.14 |
| MORPH-2 dataset | | | | |
| Gender | Front model | | | Side model |
| Female | 4.55 | | | 7.14 |
| Male | 4.84 | | | 7.22 |
| IMDB-Clean dataset | | | | |
| Gender | Front model | | | Side model |
| Female | 9.26 | | | 9.66 |
| Male | 9.17 | | | 10.5 |

**Supplementary Table S6**

| **Ethnicity** | **Gender** | **(MAE) Front Model** | **(MAE) Side Model** | **(MAE) Front+Side Model** | **(MAE) Combined Model** |
| --- | --- | --- | --- | --- | --- |
| **Black** | **Female** | 5.053439 | 5.497872 | 4.376566 | 4.538616 |
| **Black** | **Male** | 3.099434 | 3.728247 | 3.021018 | 2.881205 |
| **Hispanic** | **Female** | 4.187938 | 4.470757 | 3.936877 | 3.645679 |
| **Hispanic** | **Male** | 2.907662 | 3.473125 | 2.796301 | 2.751583 |
| **Other** | **Female** | 3.159668 | 2.944872 | 3.644901 | 2.261841 |
| **Other** | **Male** | 4.464839 | 5.734878 | 4.499807 | 4.270801 |
| **White** | **Female** | 3.496611 | 3.959518 | 3.39265 | 3.14779 |
| **White** | **Male** | 2.949113 | 3.533019 | 2.884171 | 2.747804 |

**Supplementary Table S7**

|  | Ethnic specific black | Ethnic specific Hispanic | Ethnic specific white | Full model |
| --- | --- | --- | --- | --- |
| ethnicity |  |  |  |  |
| Black | 4.245678 | 4.742421 | 6.049526 | 3.916488 |
| Hispanic | 4.562988 | 3.578347 | 3.817572 | 3.458658 |
| White | 6.068159 | 4.519732 | 3.596284 | 3.573843 |
